# Development of Highly Organized Lymphoid Structures in Buruli Ulcer Lesions after Treatment with Rifampicin and Streptomycin

**DOI:** 10.1371/journal.pntd.0000002

**Published:** 2007-10-31

**Authors:** Daniela Schütte, Alphonse Um-Boock, Ernestina Mensah-Quainoo, Peter Itin, Peter Schmid, Gerd Pluschke

**Affiliations:** 1 Swiss Tropical Institute, Molecular Immunology, Basel, Switzerland; 2 Leprosy Relief Emmaus-Switzerland (ALES) Cameroon, Yaounde, Cameroon; 3 Ghana Health Service, Amasaman, Ghana; 4 University Hospital Basel, Department of Dermatology, Basel, Switzerland; 5 Novartis Institutes of BioMedical Research, Basel, Switzerland; University of Tennessee, United States of America

## Abstract

**Background:**

Buruli ulcer caused by *Mycobacterium ulcerans* is an infection of the subcutaneous tissue leading to chronic necrotising skin ulcers. The pathogenesis is associated with the cytocidal and immunosuppressive activities of a macrolide toxin. Histopathological hallmark of progressing disease is a poor inflammatory response despite of clusters of extracellular bacilli. While traditionally wide excision of the infected tissue was the standard treatment, provisional WHO guidelines now recommend an eight week pre-treatment with streptomycin and rifampicin.

**Methodology/Principal Findings:**

We conducted a detailed immunohistochemical analysis of tissue samples from Buruli patients who received antibiotic treatment. Cellular immune response along with bacterial load and distribution were monitored. We demonstrate that this treatment leads to the development of highly organized cellular infiltration surrounding areas of coagulative necrosis. Diffuse infiltrates, granulomas and dense lymphocyte aggregation close to vessels were observed. Mycobacterial material was primarily located inside mononuclear phagocytes and microcolonies consisting of extracellular rod-shaped mycobacteria were no longer found. In observational studies some patients showed no clinical response to antibiotic treatment. Corresponding to that, one of five lesions analysed presented with huge clusters of rod-shaped bacilli but no signs of infiltration.

**Conclusions/Significance:**

Results signify that eight weeks of antibiotic treatment reverses local immunosuppression and leads to an active inflammatory process in different compartments of the skin. Structured leukocyte infiltrates with unique signatures indicative for healing processes developed at the margins of the lesions. It remains to be analysed whether antibiotic resistance of certain strains of *M. ulcerans*, lacking patient compliance or poor drug quality are responsible for the absent clinical responses in some patients. In future, analysis of local immune responses could serve as a suitable surrogate marker for the efficacy of alternative treatment strategies.

## Author Summary

Buruli ulcer (BU) is a debilitating disease of the skin presenting with extensive tissue destruction and suppression of local host defence mechanisms. Surgical removal of the affected area has been the standard therapy until in 2004 WHO recommended eight weeks' treatment with the anti-mycobacterial drugs rifampicin and streptomycin. We performed a detailed histological analysis of the local immune response in biopsies from five children medicated according to WHO provisional guidelines. One patient still revealed all histopathological signatures of an active BU lesion with huge bacterial clusters in areas of fatty tissue necrosis. Different factors can contribute to treatment failure, such as poor patient compliance and resistant bacterial strains. In four patients, different compartments of the skin presented active immune processes with only limited residues of bacterial material persisting. We demonstrated that antibiotic treatment not only directly controls the infectious agent but is also associated with fulminant host immune responses. Characterization of the healing process in BU due to therapy is highly relevant to increase our knowledge of the impact of treatment strategies to fight the disease.

## Introduction

Buruli ulcer (BU) caused by *Mycobacterium ulcerans* is a chronic necrotizing skin disease mainly affecting subcutaneous and adipose tissue [1,2]. The unique pathology of BU is primarily attributed to a plasmid-encoded macrolide toxin, mycolactone [3,4]. Mycolactone has cytopathic and apoptotic activity and is thought to be responsible for local immunosuppression by destroying infiltrating cells [3,^5^,6]. In animal models, injection of purified mycolactone causes lesions similar to those produced by wild type *M. ulcerans* bacteria [3,7].

BU is considered to be the third most common mycobacterial infection after tuberculosis and leprosy. Clinical lesions usually start as painless subcutaneous nodules that may develop into plaques or oedema. If left untreated, extensive ulcerations with typical undermined edges of the dermis develop. Spontaneous healing can occur, often leaving the patient behind with extensive scarring, retractions and deformities [8–10]. BU has been reported in more than 30 countries worldwide, but rural communities in Western and Central Africa are the worst affected [2]. Areas endemic for BU are associated with stagnant or slow-flowing water bodies. The mode of transmission is not clear; both contamination of wounds from environmental reservoirs, such as bio films on aquatic vegetation [11] and infection through the bite of insect vectors [12–14] have been discussed.

Until recently, surgery has been the only WHO recommended treatment for BU [15–17]. Wide excision margins reaching into the healthy tissue are necessary to prevent recurrences [18] and often subsequent skin grafting is required. In most endemic areas access to surgery is very limited for the majority of BU patients. Moreover, the costs for treatment and prolonged hospital stays are often prohibitive. In 2004, WHO published provisional guidelines recommending treatment with a combination of rifampicin and streptomycin [19] based on results of a small randomised controlled clinical trial [20] and observational studies. While no antibiotic therapy has been formally proven effective in BU [17], there is evidence that treatment with a combination of rifampicin and streptomycin reduces recurrence rates and may help to avoid surgery or at least limit its extent [21]. More than 50% of BU cases are children below 15 years. Potential long-term side effects of streptomycin in this population restrict the duration of the antibiotic treatment to eight weeks. If surgery is combined with antibiotic therapy, the aim is to use minimal surgery to excise necrotic tissue when antibiotics have arrested progress of the disease. For yet unknown reasons, a proportion of BU patients seem to be refractory for antibiotic treatment.

**Figure 1:**
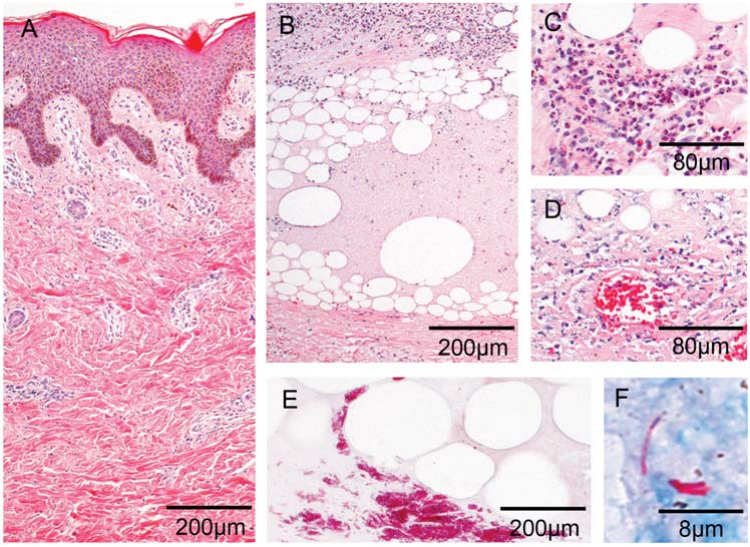
Histopathological characteristics associated with untreated Buruli ulcer lesions. Histological sections of specimen from untreated Buruli ulcer lesions stained with HE (A to D) and ZN (E, F), respectively. Photographs are taken at magnification ×40 (A, B, E), ×100 (C, D) or ×1000 (F). (A) Vasculitis associated minor leukocyte infiltration around vessels with intact dermal connective tissue and epidermal hyperplasia. (B) Extensive areas of necrosis in the deeper dermis and large fat cell ghosts with slight leukocyte infiltrates. (C) Slight cellular infiltration mainly composed of PMNL. (D) Ongoing necrotic/apoptotic processes surrounding a focus of mycobacterial microcolonies. (E) Typical clusters of extracellular bacteria between adipose cell ghosts. (F) Sometimes also single bacilli can be spotted inside necrotic regions.

Histopathological hallmarks of progressing BU are a poor inflammatory response and growing regions of necrosis of the dermal and adipose tissue eventually leading to the collapse of the overlying epidermis (Figure 1; A to D). Clusters of extracellular, mycolactone-producing acid-fast bacilli are usually located within these necrotic areas (Figure 1; E and F) [18, 22–24]. Granulomatous responses in the dermis and panniculus have been described in late stages of BU [18,^22^,25]. Observations both in cell culture and rodents experimentally infected with mycolactone producing and mycolactone-negative *M. ulcerans* strains indicate that infiltrating cells are killed due to the cytotoxic and apoptosis inducing activity of mycolactone [5,^26^,27]. While *M. ulcerans* may be captured by phagocytes during different stages of infection, it appears to persist only transiently inside these host cells [6,28]. After killing of the phagocytes, extracellular growth leads to the development of extracellular bacterial foci in areas of coagulating necrosis [18,27]. The aim of the present study was to analyse whether the local immunosuppression in Buruli ulcer lesions can be reversed by the combination treatment with rifampicin and streptomycin and if intralesional cellular immune responses complement antibiotic therapy.

## Materials and Methods

### Clinical specimens

Surgical specimens from five patients aged between six and 11 years with ulcerative lesions were obtained from the Amasaman Health Centre in Ghana and the Ayos district hospital in Cameroon (Table S1). Lesions were not older than three months and located at the lower leg (four patients) or arm (one patient). Patients had received the currently recommended standard treatment comprising surgical excision of lesions after pre-treatment with a combination of rifampicin and streptomycin (WHO, 2004). Although no further ulcer enlargement and reduction of oedema were observed, the responsible clinicians had decided to treat all five patients surgically to remove necrotic tissue and facilitate wound healing. After receiving informed consent from the guardians of the patients, surgical specimens were used for laboratory reconfirmation of clinical diagnosis and for detailed immunohistochemical analysis. All five specimens were positive for at least two of three diagnostic tests applied (Buruli ulcer-Diagnosis of *Mycobacterium ulcerans* disease, WHO, 2001), IS2404 PCR, microscopic detection of acid-fast bacilli and observation of characteristic histopathological changes. After receiving informed consent the immunologically non responding patient was tested for HIV positivity. Ethical approval for analysing patient specimens was obtained from the ethical review board of the Noguchi Memorial Institute for Medical Research and the National Ethics Committee of Cameroon.

### Immunohistochemistry

Immediately after surgery, specimens with a volume of about 0.5 cm^3^ were collected from different areas of the excised lesions to characterize the gradient of histopathological changes from necrotic areas to healthy appearing tissue at the excision margins (Figure S1). Tissue samples were fixed overnight in neutral buffered 4% paraformaldehyde, transferred to 70% ethanol, embedded in paraffin according to standard protocols and cut into 5 µm sections using a microtome. After deparaffinization sections were rehydrated through graded alcohols, endogenous peroxidase was blocked with 0.3% H_2_O_2_ for 20 min and unspecific binding prevented by incubating with blocking serum matching the secondary antibody host (Table 1). Antigen retrieval treatment was performed according to standard protocol (Dako). After antigen retrieval antibodies (Table 1) were diluted in PBS containing 0.1% Tween-20 and slides incubated for 1 h at room temperature under rocking conditions. Afterwards sections were incubated for 30 min with a correspondent biotin-conjugated secondary antibody (Table 1) and for another 30 min with streptavidin-horseradish peroxidase conjugate (VECTASTAIN ABC Kit, Vector Laboratories). Staining was performed using Vector NovaRED and haematoxylin (counterstain). Slides were subsequently mounted with Eukitt mounting medium.

**Table 1:** Antibodies used for immunohistochemistry

target antigen	stained cell type(s)	host (clone)	working dilution	retrieval method	source
CD1a	Langerhans cells	mouse (O10)	prediluted	Citrat	Beckman Coulter
CD3	T lymphocytes	rabbit	1/100	Citrat	Dako
CD4	T helper lymphocytes	mouse (1F6)	1/100	EDTA	Novocastra
CD8	T cytotoxic lymphocytes	mouse (4B11)	1/100	EDTA	Novocastra
CD14	Phagocytes	mouse (7)	1/50	Citrat	Novocastra
CD20	B lymphocytes	mouse (7D1)	1/100	Citrat	Novocastra
CD45RO	Activated lymphocytes	mouse (UCHL1)	1/100	none	Dako
CD56	Natural Killer cells	mouse (SPM489)	1/100	Citrat	Lab Vision
CD68	Antigen presenting cells	mouse (KP1)	1/50	Trypsin	Dako
elastase	Neutrophilic leucocytes	mouse (NP57)	1/50	none	Dako
Ki67	Proliferating cells	rabbit	1/50	Citrat	Dako
S100	Dermal dendrocytes	rabbit	1/400	Trypsin	Dako
mycobacterial antigens	Mycobacteria	pAbLep mouse antiserum	1/1000	Citrat	Leprosy Research Support
biotinylated anti-rabbit IgG	Secondary antibody	goat	1/200	-	Vector
biotinylated anti-mouse IgG	secondary antibody	horse (rat adsorbed)	1/200	-	Vector

Staining with Ziehl Neelsen (ZN) and Haematoxylin/Eosin (HE) was performed on all collected tissue specimen. Staining for acid-fast bacteria was performed according to WHO standard protocol (WHO Diagnostic booklet). In brief, sections were deparaffinized and rehydrated followed by incubation with ZN carbolfuchsin for 30 min at RT. Subsequently slides were washed in cool tap water for 5–10 min and individually differentiated with acid-alcohol. Counterstain was completed with haematoxylin and slides were mounted with Eukitt mounting medium.

Pictures taken with a Nikon optiphot-2 microscope were saved using analySIS soft imaging system and processed with Adobe Photoshop CS.

## Results

### Lack of a marked inflammatory response and abundant clusters of extracellular bacilli in one of five antibiotic treated patients

**Figure 2:**
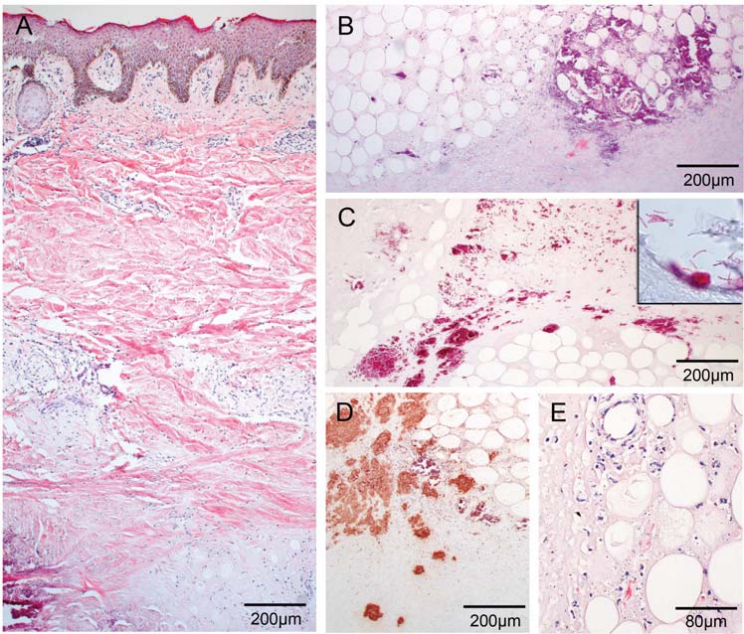
One patient exhibits strong histopathological signs for a progressive Buruli ulcer and large mycobacterial clumps. Histological sections of the non-responding patient stained with HE (A, B, D) and ZN (C) or polyclonal anti-leprae antibody (pAbLep; E). Magnification ×40 (A, B, C, E) and ×100 (D). (A) Tissue shows typical signs of advanced Buruli ulcer as deep dermal necrosis, calcification or epidermal hyperplasia. Leucocytes are found in rare cases around vessels and hardly ever between fat ghosts. (B) Adipose tissue and its surroundings are highly necrotic and happen to accumulate fat ghosts and calcification. (C) The centre of the necrotic lesion harbours tremendous clumps of rod-shaped mycobacteria. (D) Staining with pAbLep demonstrates the focal clusters of live bacteria in the necrotic core. (E) If cellular infiltration occurs in small amounts within some parts of the intradermal adipose tissue cells display apoptotic features.

The excised lesion of one of the five antibiotic treated patients was almost devoid of cellular infiltrates. Only minor superficial vasculitis-associated infiltrates of the dermis consisting of lymphocytes and macrophages/monocytes were found (Figure 2A). Extensive connective tissue necrosis and epidermal hyperplasia was observed (Figure 2A). The adipose regions displayed fat cell ghosts, extensive calcification and massive necrosis, associated with nearly complete absence of intact cell structures (Figure 2B; calcified areas stained purple). While the ulcerative centre of the lesion harboured numerous large clusters of rod-shaped extra-cellular acid fast bacilli (Figure 2C), margins contained only very few small bacterial microcolonies (not shown). A polyclonal antiserum, raised against *M. leprae* and highly cross-reactive with other mycobacteria, was used to stain *M. ulcerans* for confirmation of ZN staining results. Staining of common mycobacterial antigens with polyclonal anti-leprae antibody (pAbLep) antiserum and haematoxylin counterstain revealed an accumulation of mycobacteria (Figure 2D; red-brown) close to fat cell ghosts and necrotic calcified tissue (purple). In-between fat cell ghosts leukocytes exhibiting signs of defragmentation of nuclei and loss of cytoplasm were found only sporadically (Figure 2E). Taken together, features of this lesion resembled that of specimens from untreated patients (Figure 1) [18]. The patient was tested negative for HIV.

### Highly organized inflammatory responses and intracellular bacterial material in four of five antibiotic-treated patients

**Figure 3:**
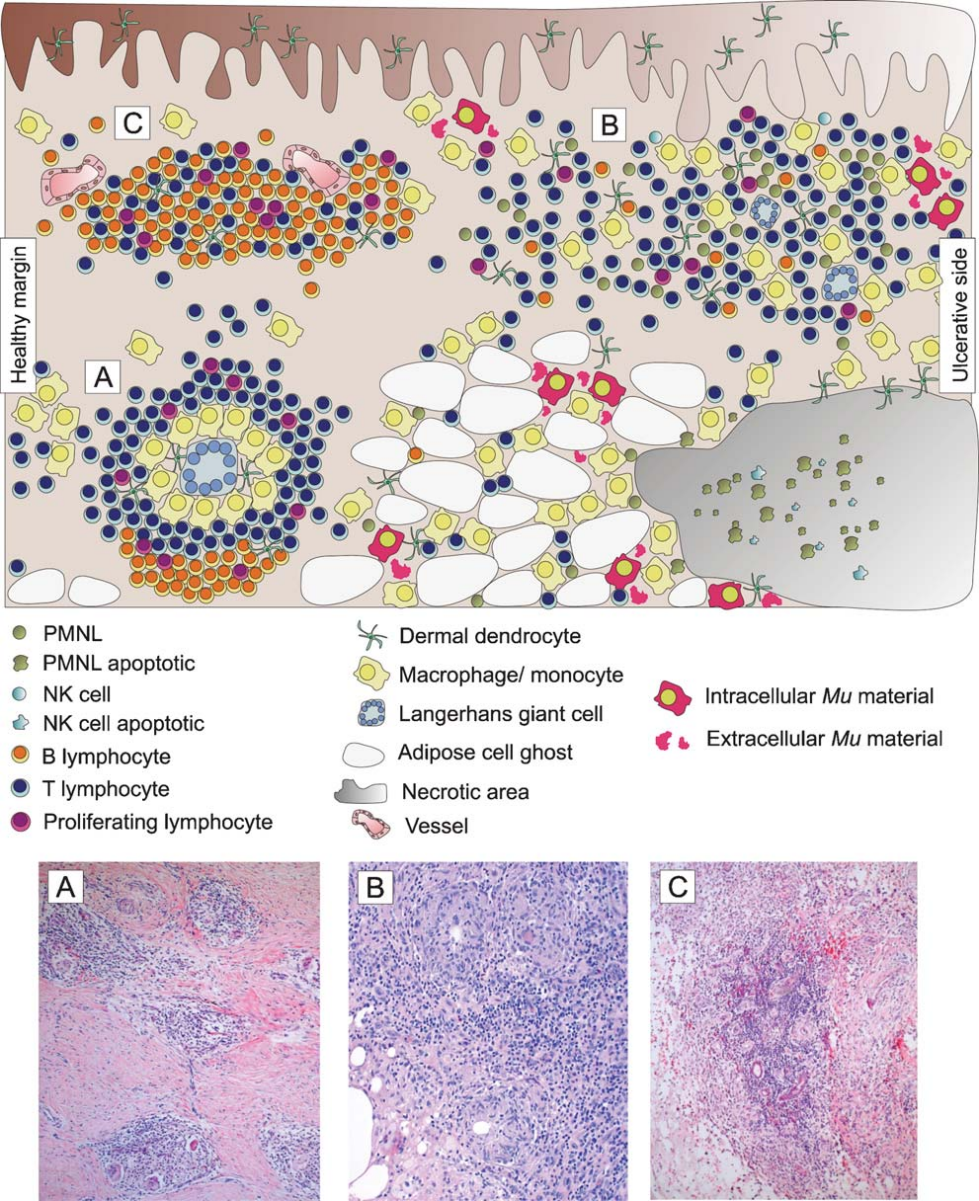
Three major types of cellular infiltration can be distinguished in antibiotic treated BU patients. Upper part: Schematic overview of cellular infiltration patterns and distribution of mycobacterial material. Lower part: Three types of cellular infiltration are documented with HE. (A) Granuloma formation in the connective tissue; magnification ×40. (B) Diffuse heterogeneous cellular infiltration of the connective and adipose tissue; magnification ×100. (C) Follicle-like lymphocyte focus adjacent to vessels; magnification ×40.

In the other four patients massive cellular infiltration was observed. Three major types of mixed infiltrates, differing in cellular composition, architecture and localisation were found in all four specimens: (i) highly organized epithelioid granulomas of different size and state of differentiation, primarily located in deeper dermal tissue (Figure 3A); (ii) less organized diffuse infiltrates representing the most abundant type, present in all areas of the dermal connective and adipose tissue (Figure 3B); (iii) dense lymphocyte clusters in proximity to vessels, occasionally found in superficial connective tissue (Figure 3C). Cellular composition and localisation of these structures along with the distribution of mycobacterial material are also schematically represented in Figure 3.

**Figure 4:**
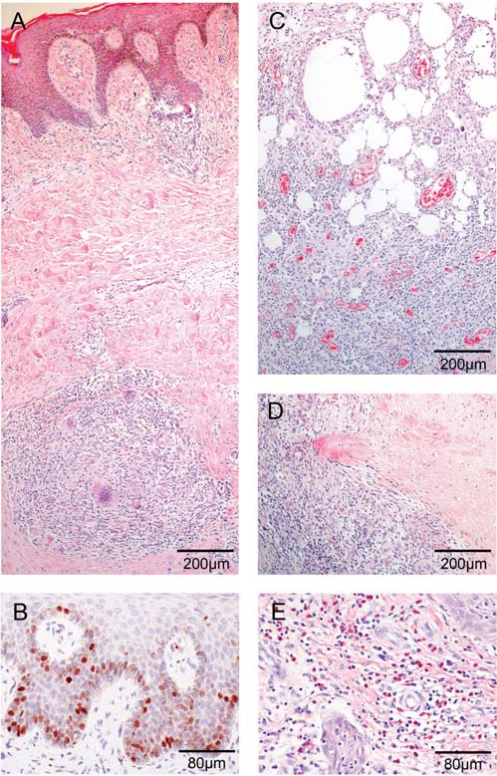
Histopathology of four patients in response to rifampicin/streptomycin bi-therapy. Histological sections representative for four patients stained with HE (A, C, D, E) and polyclonal antibody against proliferation marker Ki67 (B). Magnification ×40 (A, C, E) and ×100 (B, E). (A) Psoriatic epidermal hyperplasia typically seen in Buruli. Diffuse mixed cellular infiltrates in upper and granuloma formation with Langhans' giant cells lymphocytes in deeper dermis. (B) Ki67 staining reveals elevated proliferation levels of keratinocytes in epidermal basal layer. (C) Cell ghosts of the adipose tissue characteristic for Buruli infection. Massive mixed cellular infiltrates between fat ghosts mainly consisting of macrophages/monocytes and formation of new blood vessels. (D) Necrotic area in deep tissue encircled by extensive cellular infiltrates. (E) Focal eosinophilia found at margins of the excised area distant to ulcerative centre.

Histopathological characteristics of BU were still found in all four patients. These included psoriasiform and pseudoepitheliomatous epidermal hyperplasia and depigmentation (Figure 4A) with exceeding proliferation of keratinocytes (Figure 4B). Additionally, extensive necrosis of adipose tissue resulted in the appearance of fat cell ghosts (Figure 4C). Cellular infiltration was generally more profuse in vicinity to the necrotic centre of a lesion and declined towards the excision margins. Granulomas were composed of foamy histiocytes and Langhans' giant cells surrounded by lymphocytes and some plasma cells (Figure 4A). No central caseous necrosis was observed. In deeper adipose tissue dense infiltration with high proportions of macrophages and new blood vessel formation were observed (Figure 4C). Occasionally signs for calcification of deep dermal tissue were present (not shown). Areas of necrotic connective tissue were encircled by large accumulations of leukocytes (Figure 4D). Some leukocytes exhibiting apoptotic features resided inside necrotic regions (Figure 4D). The periphery of lesions revealed focal superficial eosinophilia (Figure 4E).

**Figure 5:**
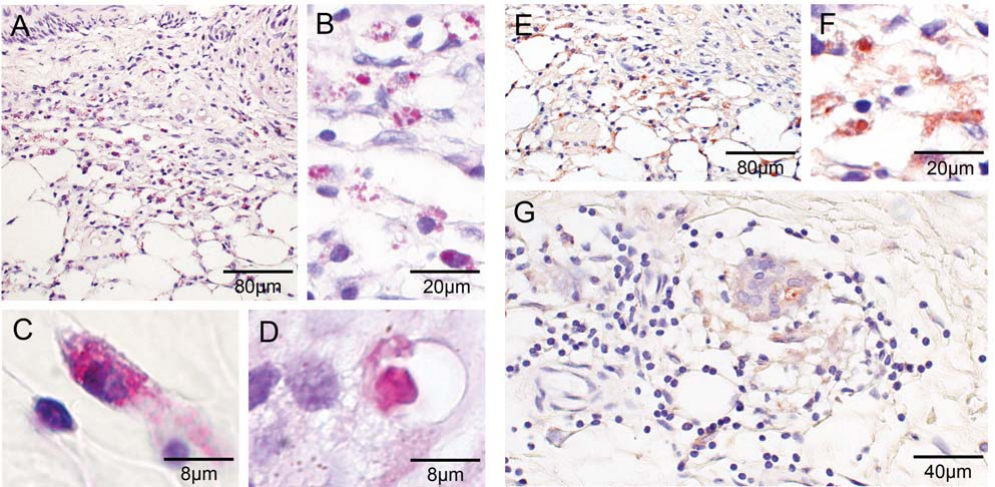
Bacterial load and distribution in four patients. Histological sections representative for four patients stained with ZN (A to D) and polyclonal anti-leprae antibody (pAbLep; E to G) to demonstrate distribution of mycobacterial material in the excised lesion. Counterstain was performed with haematoxylin and pictures taken at magnifications of ×100 (A, E), ×200 (G), ×400 (B, F) and ×1000 (C, D). (A, E) Serial sections demonstrate equal detection sensitivity with ZN and pAbLep. Higher amounts of bacterial material near the ulcerative edges with macrophages being the most prevalent leucocytes. (B, F) Close-up of A and E, respectively. Foci of rounded bacteria located extra- and intracellular between fat ghosts. (C) Macrophage presenting with mycobacterial material within phagosomal vesicles. (D) Mycobacterial residues appear in rare cases inside Langhans' giant cell phagosomal vacuoles. (G) Small granuloma with giant cell formation and traces of mycobacteria inside a phagosome.

The distribution of mycobacterial material was assessed by ZN staining (Figure 5A–5D) and immunostaining with pAbLep antiserum (Figure 5E–5G). Both staining methods were found to be equally sensitive when compared in serial sections (Figure 5A and 5B vs. 5E and 5F). In all four patients mycobacterial material was predominantly found inside macrophages, although some was still located extra-cellularly; both extra- and intracellular bacilli had lost their characteristic rod-shape appearance (Figure 5B and 5F). While the cytoplasm of macrophages frequently harboured numerous phagosomes containing mycobacterial material (Figure 5C), only a few Langhans' giant cells with vacuoles containing ZN and serologically stainable material were located within granulomas (Figure 5D and 5G, respectively). The highest burden of mycobacterial material was found in deep dermal regions (Figure 5A and 5E) with a declining gradient from ulcerative areas towards the excision margins. Accumulations of mycobacterial material were primarily observed in areas of mixed cellular infiltration, but only very rarely in granulomas. Additionally, few residues of microcolonies were present in the upper dermal connective tissue or close to the epidermal basal layer (not shown).

### Architecture of granulomas

**Figure 6:**
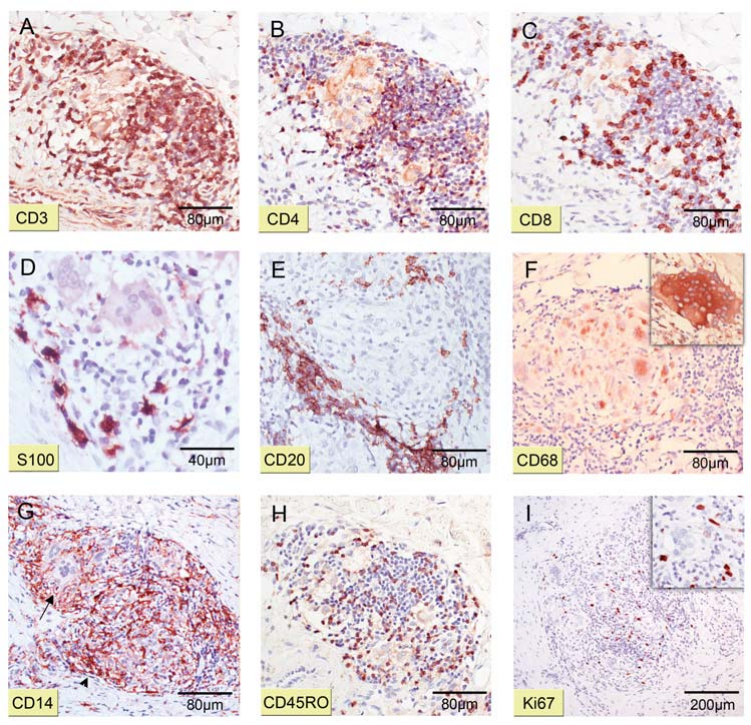
Detailed organization of granulomas. Histological serial sections representative for four patients were stained with antibodies against different cellular surface or cytoplasmic markers (counterstain haematoxylin). Magnification ×40 (I), ×100 (A, B, C, E, F, G, H, I_insert_), ×200 (D) and ×400 (F_insert_). (A, B, C) Staining with CD3, CD4 and CD8, respectively, reveals a belt of helper as well as cytotoxic T lymphocytes surrounding the APC core. (D) S100^+^ dermal dendrocytes (dDC) spread among T lymphocytes in the outer layer of a granuloma. (E) Focus of CD20^+^ B lymphocytes at the border of a granuloma. (F) CD68^+^ APC in the centre of a representative granuloma; insert shows large Langhans' giant cell. (G) Remarkable large amounts of membrane bound (arrow) and soluble (arrowhead) CD14 can be observed. (H) Distribution of activated CD45RO^+^ lymphocytes. (I) Proliferating Ki67^+^ cells indicate the active status of granulomas.

A panel of antibodies specific for leukocyte markers (Table 1) were used for immunohistochemical characterisation of infiltrates. Serial sections revealed that the outer layer of granulomas was mainly composed of CD3^+^ T lymphocytes (Figure 6A) with CD4^+^ T cells (Figure 6B) always outnumbering CD8^+^ T cells (Figure 6C). These lymphocyte belts were interspersed with S100^+^ dermal dendrocytes (dDCs) (Figure 6D). Occasionally dDCs were present in the centre of granulomas (not shown). Focal clusters of CD20^+^ B cells appeared at the outer margins of the T lymphocyte layer (Figure 6E). Cytoplasmic CD68^+^ antigen-presenting cells (APCs) in particular Langhans' giant cells (Figure 6F_insert_) and epithelioid macrophages formed the centre of granulomas (Figure 6F). Staining of the membrane protein CD14 confirmed the macrophage/monocyte origin of giant cells (Figure 6G, arrow). Furthermore, large amounts of soluble CD14 were observed within the belt of T lymphocytes (Figure 6G, arrowhead). A great proportion of lymphocytes turned out to be activated as evidenced by their CD45RO^+^ phenotype (Figure 6H). Variable numbers of Ki67^+^ (proliferating) cells (Figure 6I) presented a lymphocyte phenotype (Figure 6I_insert_). Neither elastase^+^ polymorphonuclear neutrophilic leucocytes (PMNL) nor CD56^+^ natural killer (NK) cells could be detected in granuloma formations.

### Cellular composition of diffuse mixed infiltrates

**Figure 7:**
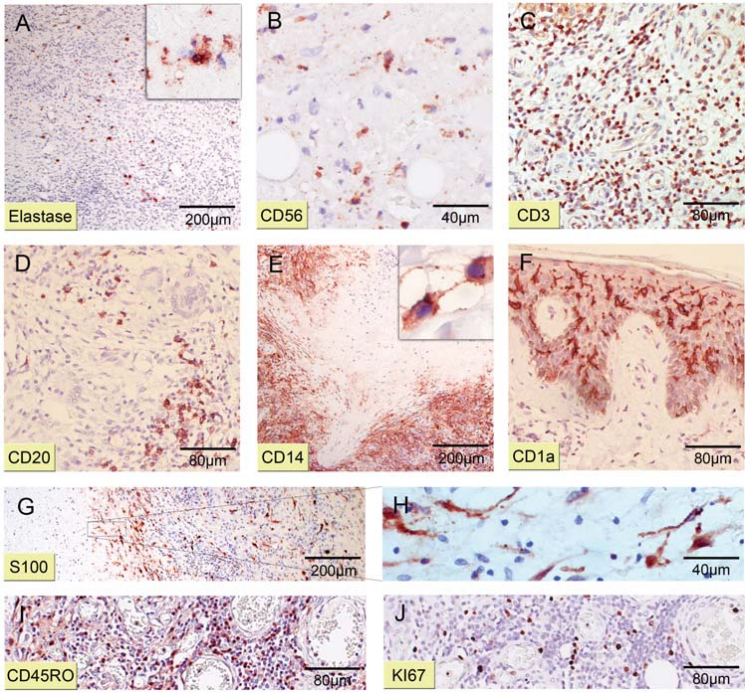
Composite of mixed cellular infiltrates. Histological sections representative for four patients stained with antibodies against different cellular markers (counterstain haematoxylin). Magnifications at ×40 (A, E, G), ×100 (C, D, F, I, J), ×200 (A_insert_, B, H) or ×1000 (E_insert_). (A) Only few scattered PMNL staining positive for Elastase were found within cellular infiltrates. (A_insert_, B) Neutrophilic and NK cell (CD56^+^) foci inside necrotic areas display signs of advanced apoptosis. (C) Lymphocytes mainly expose a CD3^+^ phenotype. (D) Small focal CD20^+^ lymphocyte spots are scattered through infiltrates. (E) Staining against CD14 illustrates large numbers of histiocytes enclosing necrotic tissue shedding massive amounts of sCD14 (insert). (F) Levels of epidermal CD1a^+^ Langerhans cells are remarkably elevated compared to healthy skin. (G) Aggregation of S100^+^ dDCs near necrotic spots. (H) Elongated cellular appendices of dDCs reach into the necrotic tissue. (I) Large numbers of lymphocytes are CD45RO^+^. (J) Same area as in (I). Proliferating lymphocytes are highly Ki67^+^.

In contrast to granulomas, regions of diffuse cellular infiltration contained sparsely distributed elastase^+^ PMNL (Figure 7A) with sporadic focal clusters near ulcerative and necrotic areas (not shown). CD56^+^ NK cells showed a similar distribution with even lower cell counts (not shown). Foci of PMNL and NK cells with signs of apoptosis were located within necrotic tissue (Figure 7A_insert_ and 7B, respectively). CD3^+^ T lymphocyte (Figure 7C) and foamy histiocytes (not shown) were the most prominent cell types of mixed infiltrates in the dermal connective tissue. The CD4^+^/CD8^+^ cell ratio varied widely between different regions of a lesion, but usually CD8^+^ cells were more abundant than CD4^+^ cells (not shown). Small clusters of CD20^+^ B lymphocytes were scattered within infiltrates (Figure 7D). Counts of CD14^+^ macrophages/monocytes were particularly high in adipose tissue and around necrotic areas (Figure 7E). Additionally, vast amounts of sCD14 shed from those cells were revealed (Figure 7E_insert_). Dendritic cells could be detected in both epidermis and dermis. The frequency of epidermal CD1a^+^ Langerhans cells was strongly elevated (Figure 7F). S100^+^ dDC were distributed throughout diffuse mixed infiltrates with increasing density towards the margins of necrotic areas (Figure 7G). Remarkably, dendritic cell appendices reached into the damaged tissue (Figure 7H). Similar to the findings in granulomas, a large proportion of lymphocytes stained positive for the activation marker CD45RO (Figure 7I) and proliferating Ki67^+^ cells were scattered throughout the infiltrate (Figure 7J).

### Aggregates of lymphocytes resembling follicular structures

**Figure 8:**
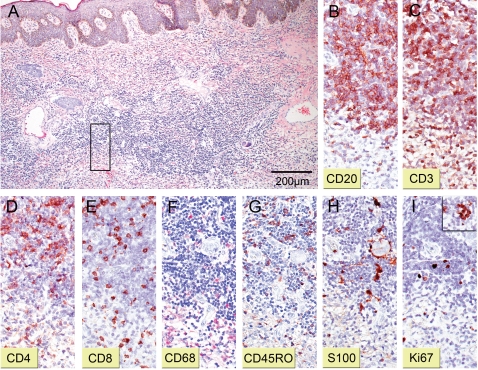
Follicle-like lymphocyte foci reveal organized cellular formation. Serial sections of a representative lymphocyte focus stained with HE (A; magnification ×40) or with antibodies against different cellular markers and haematoxylin as counterstain (B to I; magnification ×100). (A) Large aggregate of lymphocytes in the upper dermal layer between two venules. Square marks area of magnification chosen for pictures B to I. (B) CD20+ B lymphocytes build the most abundant cell subset. (C, D, E) Staining against CD3, CD4 and CD8, respectively, exposes also high loads of T lymphocytes with T helper clearly being more prevalent than cytotoxic T cells. (F) APCs (CD68+) are scattered throughout the entire structure. (G) Activated lymphocytes appear positive for CD45RO staining. (H) S100+ dermal dendrocytes (dDC) lie in between lymphocytes. (I) Ki67 staining demonstrates certain proliferation of cells and occasional appearance of discrete spots of hyperproliferation (insert).

Adjacent to lymphatics or blood-vessels a third type of infiltration was found, i.e. follicle-like structures with dense aggregations of lymphocytes (Figure 8A). CD20^+^ B lymphocytes were the dominating cell type (Figure 8B) followed by CD3^+^ T lymphocytes (Figure 8C). Here CD4^+^ T cells (Figure 8D) were more frequent than CD8^+^ T cells (Figure 8E). Additionally, few CD68^+^ APCs (Figure 8F) and S100^+^ dDCs (Figure 8H) were scattered throughout the aggregate. Similar to the other two infiltration types, a large proportion of lymphocytes stained positive for the activation marker CD45RO (Figure 8G). Single proliferating Ki67^+^ cells were evenly distributed (Figure 8I) and sporadically hyper-proliferative clusters were encountered (Figure 8I _insert_).

## Discussion

It has been demonstrated recently that treatment with a combination of rifampicin and streptomycin inhibits the growth of *M. ulcerans* in pre-ulcerative BU lesions [19]. Furthermore, observational studies [21] indicate that at least in some of the BU patients antibiotic therapy may reduce the extent of or even circumvent the indication for surgery. Here we present results of a detailed immunohistological analysis of ulcerative BU lesions from patients treated with rifampicin/streptomycin prior to surgery. Findings were compared to those obtained from lesions of patients that have been treated merely with wide-ranging surgical excision (Figure 1) according to former WHO treatment guidelines [1,18]. To our knowledge this is the first detailed immunohistological description of the effect of antibiotic treatment on BU lesions.

Presentation of the affected tissue of four of the five antibiotic treated patients was strikingly different from that of untreated patients, in that immense leukocyte infiltrates and the formation of new vessels were observed. These findings reconfirm results of Etuaful et al., who observed an induction of chronic inflammation and granulomas in pre-ulcerative lesions by antibiotic treatment [19]. Our detailed analysis regarding composition and architecture of cellular infiltrates is indicative for the development of highly organized tertiary lymphoid tissue. Islands of infiltration and scattered granulomas may also develop in untreated late stage lesions [18,^22^,25]. However, infiltration in the patients at hand was much more substantial, resembling histopathological patterns observed in conditions such as dermatoborreliosis, where lymphoid neogenesis is reported [29]. We found three different types of infiltration in all four responding patients (Figure 3): granulomas, diffuse mixed infiltrates and dense lymphocyte aggregations in the vicinity to vessels. We assume that this clear structural differentiation reflects a range of different functional activities required for complete clearance of infection and resorption of necrotic tissue.

In regions of diffuse infiltration, in particular close to areas of necrosis, outstandingly large amounts of both membrane-bound and soluble CD14 were found. Consistent with this, it has been reported that the development of highly organized structures, such as granulomas, is not required for the resorption of destroyed tissue [30]. CD14 mediated clearance of necrotic tissue is usually not associated with an increased expression of inflammatory cytokines [30], which is in line with the observed lack of major inflammatory symptoms in the enrolled patients. Necrotic regions were surrounded by a substantial quantity of dDCs and their appendices reached into the damaged tissue, indicating enhanced antigen uptake and presentation. Moreover, elevated numbers of Langerhans cells were distributed throughout the epidermis, like it has been described for tuberculoid and borderline leprosy [31]. A large proportion of lymphocytes were expressing activation and proliferation markers. Taken together, these observations reveal that adaptive immune responses are taking place autonomously.

While in human tuberculosis granulomas develop a central necrotic core [32], this was not observed in the lesions of the antibiotic treated BU patients. Here, the centre was mainly formed of foamy histiocytes and Langhans' giant cells, like reported for leprosy [33], and mycobacterial material was only infrequently detected. In leprosy, cells of the lymphocyte belt are less tightly packed [34] than in tuberculosis [35], a feature we also observed in BU lesions. Thus, BU granulomas may function primarily as a place for antigen presentation and adaptive immune response rather than for sequestration of the mycobacteria.

In diffuse infiltrates the CD8/CD4 T lymphocyte ratio was higher than in granulomas. Otherwise, the cellular composition of both types of infiltration was largely the same. Small islets of Langhans' giant cells inside the diffuse infiltrates support the hypothesis that granulomas represent a more advanced state of the initially unorganized infiltrations. In contrast, the dense lymphocyte aggregations we observed in vicinity to vessels were of a markedly different cellular composition. Here B lymphocytes represented the most dominant cell type in contrast to granulomas or unorganized infiltrates, where small B cell clusters were distributed more sparsely. B and T lymphocytes were packed in a notably dense manner, with interspersed dDCs and APCs and no central core, a composition characteristic also for secondary lymphoid organs. Similar structures have also been reported by Ulrichs et al in lung tissue from tuberculosis patients [35]. Many lymphocytes were in the activated state as demonstrated by CD45RO staining and a substantial proportion displayed the proliferation marker Ki67. It has been suggested that these cell aggregations represent active centres, which orchestrate the local host defence [35]. It is not clear, which factors play a crucial role in the development of ectopic lymphoid tissue induced via antibiotic treatment. Plasmacytoid and myeloid dendritic cells present in BU lesions prior to antibiotic treatment [36] may play an important role in this process. Lymphangiogenesis and participation of lymphoid tissue-inducer cells may represent hallmarks in the process of lymphoid neogenesis [29]. Although tertiary lymphoid organs seem to develop in infectious diseases to sequester pathogens, this process is often accompanied by tissue damage [29]. Anecdotal reports on the emergence of new ulcerations in the course of antibiotic treatment of BU, probably at sites containing unrecognized infection foci, may be a hint into this direction. It remains to be analysed whether in some cases antibiotic treatment may even accelerate the progression of early plaques and oedemas to ulcerative lesions.

In animal infection models neither wild type nor mycolactone negative *M. ulcerans* strains were strong neutrophilic attractants [6], although other studies reported substantial neutrophilic infiltrates in early stage infections [28]. Infiltrates in patients treated with antibiotics contained only very low levels of neutrophilic leukocytes. In contrast, large numbers of apoptotic cells positive for the neutrophilic leukocyte marker elastase and the NK cell marker CD56 were present within necrotic regions. Findings with an experimental *M. ulcerans* mouse infection model [27] indicate that these apoptotic cells represent the residues of an early acute defence line. Our data show for the first time that cells of this early defence are destroyed together with the surrounding tissue, if a substantial infection focus develops. It is most likely that the generalized cytotoxic activity of mycolactone is the all-dominant factor.

Lesions of all four responders still harboured mycobacterial material as revealed by ZN and immunohistochemical staining. However microcolonies consisting of extracellular [9],[22] or intracellular [37] rod-shaped mycobacterial cells were no longer found. Stainable mycobacterial material had lost the characteristic shape and was primarily located inside mononuclear phagocytes, but was also present in small extracellular foci. These data indicate a killing or debilitation of the bacteria in the course of antibiotic treatment. Moreover, antigen uptake and presentation via macrophages and dendritic cells may be enhanced due to lower levels of mycolactone. While bacterial residues were abundant in regions of diffuse infiltration of the deeper dermis and in adipose tissue, granulomas were largely devoid of it. Only single Langhans' giant cells contained vacuoles filled with stainable residues of the mycobacterial cells. A similar pattern may be found in active pulmonary tuberculosis [35],[38]. Again, these findings support the theory that granulomas serve as a place for antigen encounter.

The density of stainable mycobacterial material increased from the outer margins of lesions towards the ulcerative centre, which is consistent with the distribution of mycobacterial DNA in untreated lesions [18],[39]. Moreover, our results demonstrate that after antibiotic treatment the mycobacteria are directly taken up by phagocytes at the original focus of infection without granuloma formation. *M. ulcerans* is able to destroy phagocytes after transient intracellular growth via the release of mycolactone [6],[28]. We think that the stainable intracellular mycobacterial material represents the remains of phagocytosed dead bacilli or of cells severely impaired in mycolactone production due to the antibiotic treatment. In general it is most likely, that a pronounced reduction of the concentration of mycolactone in the lesion is a prerequisite for the observed reversal of the local immunosuppression. Due to the lack of quantitative detection methods for mycolactone, for the time being this aspect cannot be studied.

The BU lesion of one antibiotic-treated patient displayed histopathological features similar to active lesions of patients not receiving antibiotic treatment. Only minor leukocyte infiltration was found and huge clusters of extracellular mycobacteria were located beneath the ulcerative centre. Observational studies indicate that in a certain proportion of patients, rifampicin/streptomycin treatment has no clinical curative effect. Acquired or inherited host factors, like deficiencies in the IFN-_Y_ and IL-12 pathways for macrophage activation [40],[41], may play a role. Alternatively, antibiotic resistance of certain lineages of *M. ulcerans* could be responsible for this lack of response. Therefore major efforts should be made to generate *M. ulcerans* isolates from these patients for susceptibility testing. Another factor to be considered is poor patient compliance. Rifampicin is administered once per day orally and the intake of tablets may not be monitored. Furthermore, outdated drugs and poorly prepared solutions can be possible reasons for treatment failure and clearly need to be taken into consideration.

In summary, we conclude that treatment of BU with rifampicin/streptomycin is accompanied by a reversion of the local immunosuppression. The synergistic anti-mycobacterial action of antibiotics and immune defence mechanisms may be required to clear the infection efficiently. In view of these results, it may be suitable to replace streptomycin already after a few weeks by a bacteriostatic antibiotic. Recently published data of our group indicate that not everyone exposed to *M. ulcerans* develops clinical disease [42]. After exposure a race between front line immune responses and mycobacterial multiplication seems to decide whether a chronic infection focus is established or not. Once a bacterial cluster is large enough to develop a cytocidal cloud of mycolactone around itself, necrotic areas with only minor infiltration develop [18, 22–25,43], which is in stark contrast to the vigorous immune responses we describe in the present study. We infer that a complex and highly organized cellular immune response is crucial for elimination of a chronic *M. ulcerans* infection. Efficient triggering of local immune responses may thus represent a suitable auxiliary marker for the efficacy of alternative treatment strategies.

## Supporting Information

Figure S1 Sampling of tissue for immunohistochemistry
(5.14 MB TIF)

Table S1 Main data of enrolled patients
(0.03 MB DOC)
