# Supplementary figures and images for: Development of Highly Organized Lymphoid Structures in Buruli Ulcer Lesions after Treatment with Rifampicin and Streptomycin
Source: PLoS Negl Trop Dis. 2007 Oct 31;1(1):e2. doi: 10.1371/journal.pntd.0000002 (PMC2041817; doi:10.1371/journal.pntd.0000002)

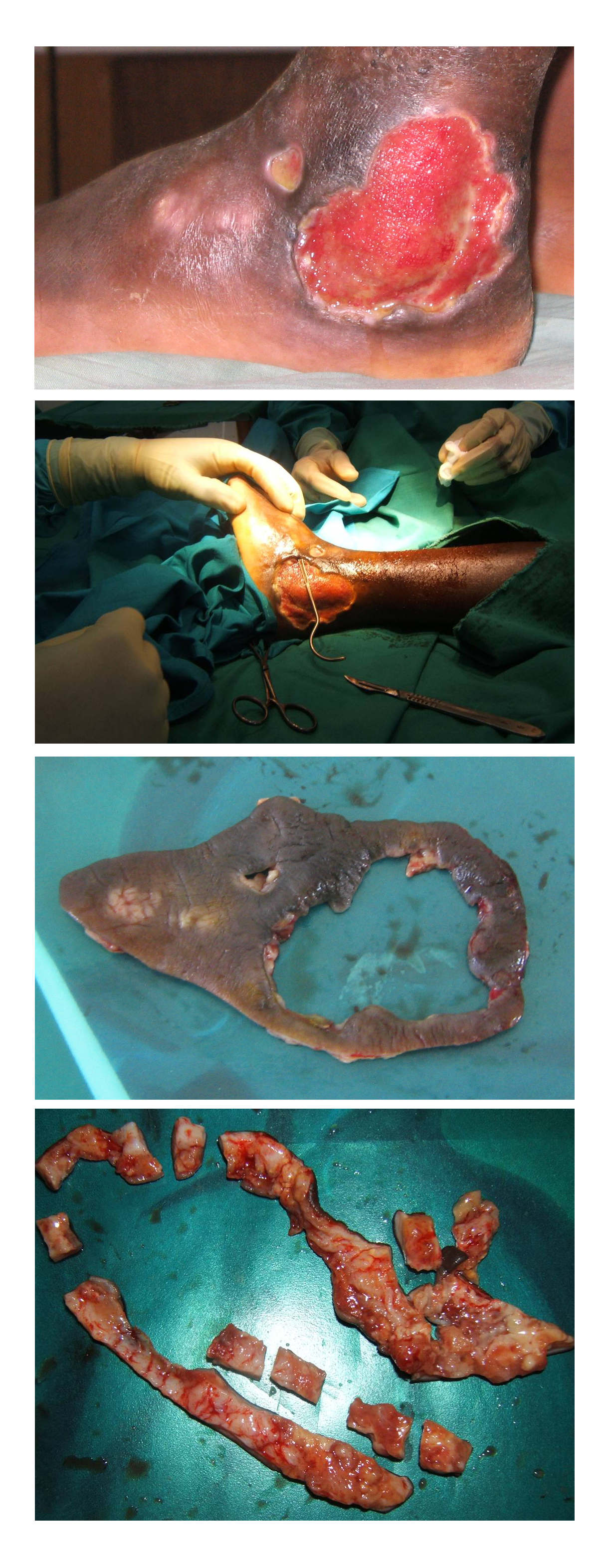

Supplement: Figure S1 — Sampling of tissue for immunohistochemistry (5.14 MB TIF) [file pntd.0000002.s001.tif]

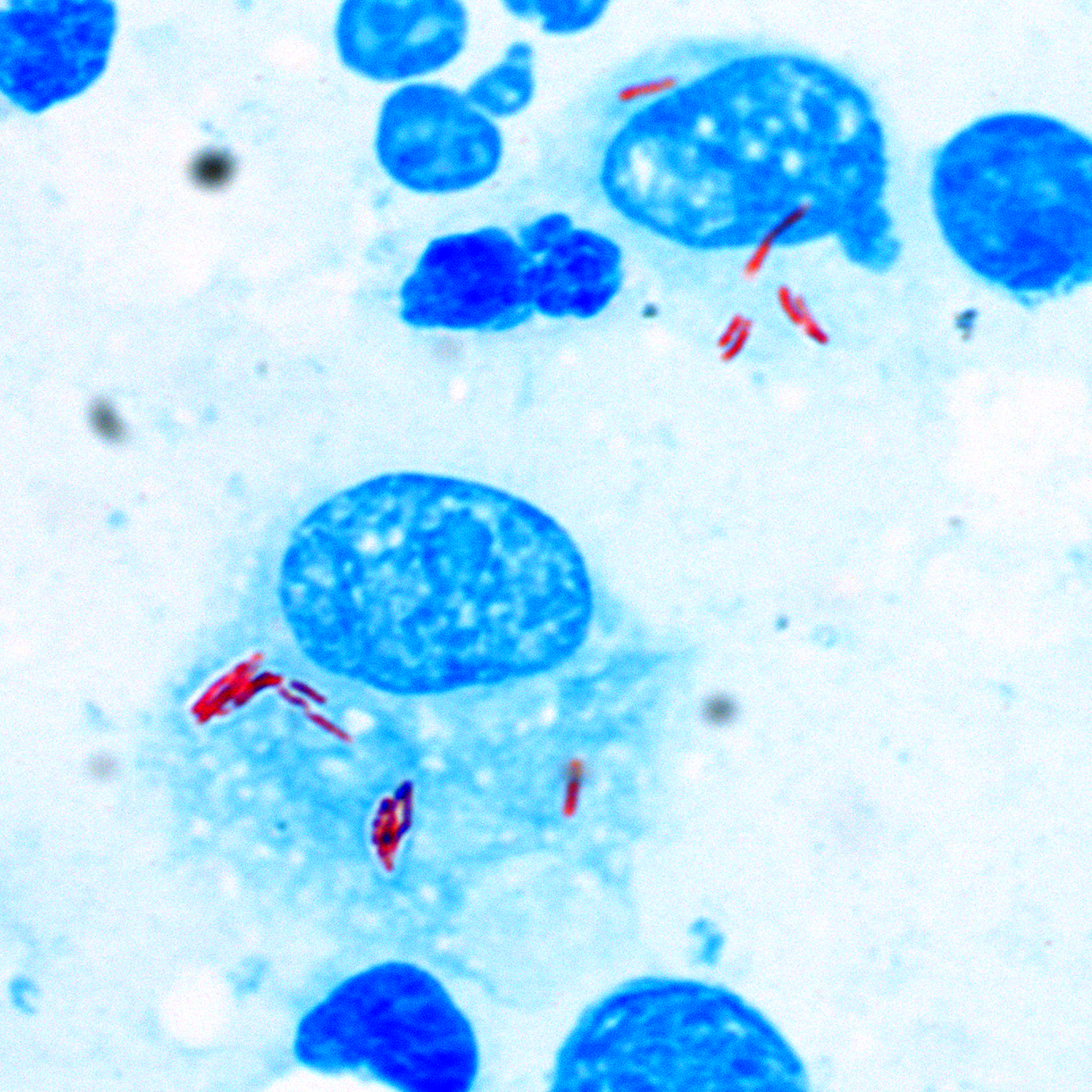

Supplement: Table S1 — Main data of enrolled patients (0.03 MB DOC) [file pntd.0000002.s002.doc]
